# Predictors of the use of analgesic drugs 1 year after joint replacement: a single-center analysis of 13,000 hip and knee replacements

**DOI:** 10.1186/s13075-020-02184-1

**Published:** 2020-04-21

**Authors:** Tuomas Jaakko Rajamäki, Pia A. Puolakka, Aki Hietaharju, Teemu Moilanen, Esa Jämsen

**Affiliations:** 1 Faculty of Medicine and Health Technology, Tampere University, 33014 Tampere, Finland; 2 Department of Anaesthesia, Tampere University Hospital, Tampere, Finland; 3 Department of Neurology, Tampere University Hospital, Tampere, Finland; 4 Coxa, Hospital for Joint Replacement, Tampere, Finland

**Keywords:** Knee replacement, Hip replacement, Analgesic drug, Opioid, NSAID, Acetaminophen, Risk factors

## Abstract

**Background:**

Pain persists in a moderate number of patients following hip or knee replacement surgery. Persistent pain may subsequently lead to the prolonged consumption of analgesics after surgery and expose patients to the adverse drug events of opioids and NSAIDs, especially in older patients and patients with comorbidities. This study aimed to identify risk factors for the increased use of opioids and other analgesics 1 year after surgery and focused on comorbidities and surgery-related factors.

**Methods:**

All patients who underwent a primary hip or knee replacement for osteoarthritis from 2002 to 2013 were identified. Redeemed prescriptions for acetaminophen, non-steroidal anti-inflammatory drugs (NSAIDs), and opioids (mild and strong) were collected from a nationwide Drug Prescription Register. The user rates of analgesics and the adjusted risks ratios for analgesic use 1 year after joint replacement were calculated.

**Results:**

Of the 6238 hip replacement and 7501 knee replacement recipients, 3591 (26.1%) were still using analgesics 1 year after surgery. Significant predictors of overall analgesic use (acetaminophen, NSAID, or opioid) were (risk ratio (95% CI)) age 65–74.9 years (reference < 65), 1.1 (1.03–1.2); age > 75 years, 1.2 (1.1–1.3); female gender, 1.2 (1.1–1.3); BMI 30–34.9 kg/m^2^ (reference < 25 kg/m^2^), 1.1 (1.04–1.2); BMI > 35 kg/m^2^, 1.4 (1.3–1.6); and a higher number of comorbidities (according to the modified Charlson Comorbidity Index score), 1.2 (1.1–1.4). Diabetes and other comorbidities were not significant independent predictors. Of the other clinical factors, the preoperative use of analgesics, 2.6 (2.5–2.8), and knee surgery, 1.2 (1.1–1.3), predicted the use of analgesics, whereas simultaneous bilateral knee replacement (compared to unilateral procedure) was a protective factor, 0.86 (0.77–0.96). Opioid use was associated with obesity, higher CCI score, epilepsy, knee vs hip surgery, unilateral vs bilateral knee operation, total vs unicompartmental knee replacement, and the preoperative use of analgesics/opioids.

**Conclusions:**

Obesity (especially BMI > 35 kg/m^2^) and the preoperative use of analgesics were the strongest predictors of an increased postoperative use of analgesics. It is remarkable that also older age and higher number of comorbidities predicted analgesic use despite these patients being the most vulnerable to adverse drug events.

## Introduction

Although hip and knee replacement are performed to reduce pain and regain function in patients with late-stage arthritis [1, 2], 10–20% of patients continue to suffer from persistent pain after surgery [3, 4]. The latest studies focusing on persistent pain indicate that its major risk factors include pain catastrophizing, intensity of preoperative pain, mental health problems, knee surgery, and certain comorbidities, such as diabetes [4–7].

Persistent pain may also lead to the increased consumption of analgesics [8]. To date, however, only a limited number of large-scale studies have been conducted on the trajectories of analgesic consumption and the risk factors associated with increased analgesic consumption after surgery [9–21], even though the risks for adverse events of opioids and non-steroidal anti-inflammatory drugs increase in long-term use [22–24], especially in elderly patients with comorbidities. Motivated by the ongoing opioid prescription crisis [25], most recent studies have focused on opioid use after joint replacement and reported higher odds for prolonged postoperative opioid use in association with psychiatric disorders, preoperative opioid use, worse preoperative pain, younger age, cardiac disease, and undergoing knee (compared to hip) surgery [9–17, 26].

The analysis of persistent pain should also include non-opioid analgesics, which at present only applies to a few studies [9, 18–20]. Indeed, high levels of preoperative pain, knee surgery (compared to hip surgery), and younger age have all been associated with an increased overall consumption of analgesic drugs [9]. Furthermore, obesity, female gender, younger age, and depression are associated with an increased consumption of NSAIDs [18–20]. However, general comorbidities, such as diabetes, a risk factor for persistent pain, have only been analyzed in one previous study [9], in which no differences in overall analgesic consumption after knee replacement were reported. To the best of our knowledge, those factors related to the consumption of acetaminophen have not previously been analyzed in the literature.

In our study, we studied the use of opioids, NSAIDs, and acetaminophen in a large sample of patients undergoing primary hip or knee replacement for osteoarthritis. Our primary aim was to find out whether postoperative analgesic use is associated with patient characteristics, such as obesity or other comorbidities, or other clinical factors such as preoperative clinical state, and analgesic use. Our secondary aim was to find out whether these factors differ depending on the type of analgesic used.

## Patients and methods

### The study population

**Fig. 1:**
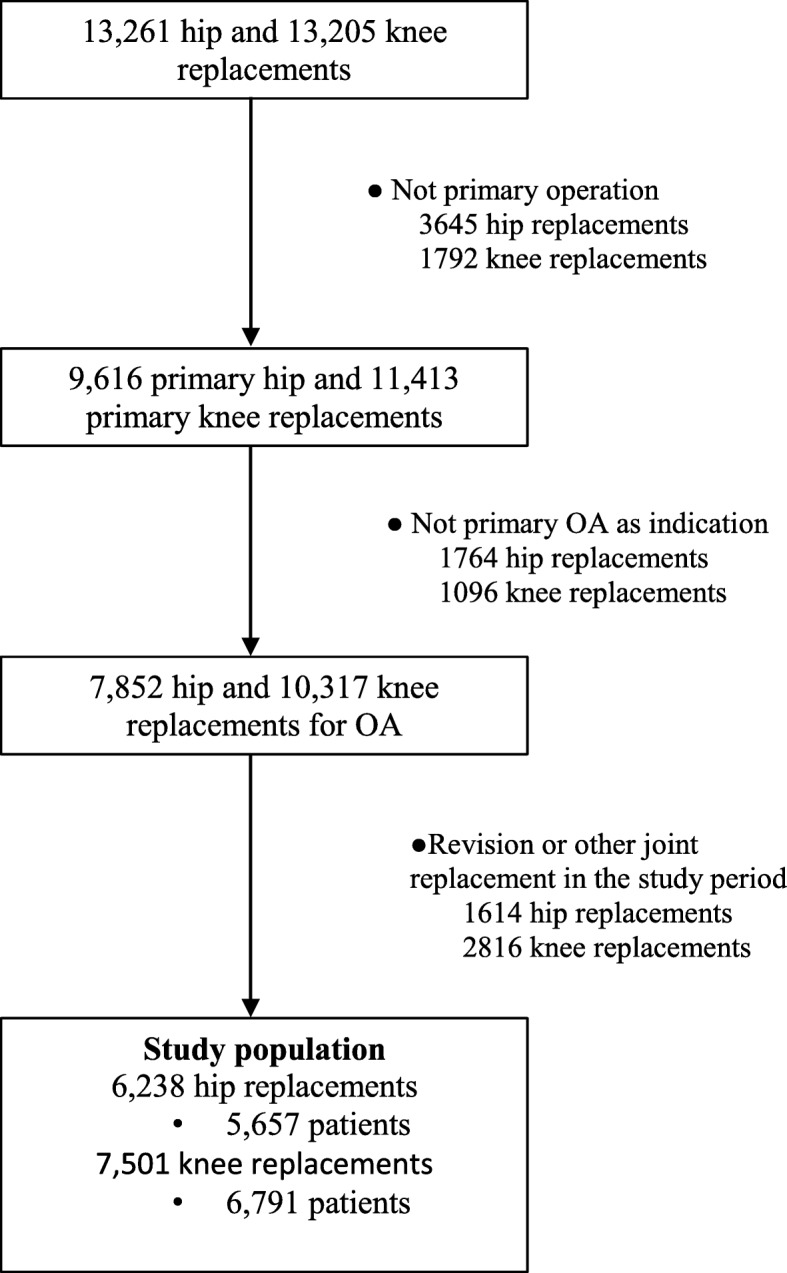
Flowchart

A total of 13,261 hip replacements and 13,205 knee replacements were performed between September 2, 2002, and December 31, 2013, in a single, publicly-funded orthopedic hospital in Finland, as reported in a previous study [27]. A prospective database of the hospital includes preoperative and postoperative clinical information on these patients. All patients who had undergone a primary operation and had primary osteoarthritis as the indication for surgery were included in this study. Patients with revisions or other joint replacements during the study period (operation date +/− 2 years) were excluded. Finally, 13,739 joint replacements (6238 hip replacements performed on 5657 patients and 7501 knee replacements performed on 6791 patients) were included (Fig. 1).

### Special reimbursement register

Finland has a health insurance system that covers all residents. A share of the costs of prescribed medications is reimbursed by the Social Insurance Institution (SII). Patients with certain severe and chronic diseases are entitled to special refunds. To be eligible for special reimbursement, a written certificate that verifies that the patient’s condition meets the predefined criteria is required from the treating physician. The SII maintains a Special Reimbursement Register that was used to extract information on chronic diseases for this study. The chronic diseases include Alzheimer’s disease, cardiac disease (coronary heart disease, heart failure, and chronic arrhythmias), hypertension, diabetes, epilepsy, malignancies, asthma or other severe obstructive pulmonary disease, severe kidney disease, Parkinson’s disease, psychoses, and rheumatic diseases.

### Medication data

In addition to the Special Reimbursement Register, the SII also maintains a nationwide Drug Prescription Register that contains information on all prescribed medications that have been dispensed from pharmacies in Finland. In this study, information on the Anatomical Therapeutic Chemical (ATC) code of the dispensed drugs, the number of units dispensed (tablets or patches), and the date of purchase was collected from the Drug Prescription Register 2 years prior to and after surgery. The analyzed analgesics were acetaminophen (N02BE01), NSAIDs (including coxibs; M01A), and opioids (N02A). Depression is a significant risk factor for persistent pain and analgesic use [4, 5, 9, 18, 19, 26], and therefore, information on antidepressant (N06A) purchases within a period of 1 year prior to surgery was registered to act as an indicator of depression because only psychotic depression is recorded in the Special Reimbursement Register. Over-the-counter (OTC) drugs, which in Finland include small packs of acetaminophen, ibuprofen, and ketoprofen, were not recorded in this study. In Finland, all medications (including OTC drugs) are sold from pharmacies.

### Statistics

The proportions of patients who redeemed at least one prescription of the studied medication were calculated in time-periods of 3 months (90 days), 2 years preoperatively, and 2 years postoperatively. The analgesic groups included opioids, NSAIDs, acetaminophen, and any analgesics (previous groups combined).

In this study, the focus was on those factors associated with the use of analgesics preoperatively (0–3 months before surgery) and 1 year postoperatively (9–12 months after surgery). The factors analyzed that were potentially associated with the use of analgesics included chronic diseases at the time of surgery (according to the Special Reimbursement Register data). In addition, clinical data which was collected at preoperative outpatient clinic visit within a few weeks before surgery were extracted from the prospective database of the hospital that included age, gender, body mass index (BMI), laterality (unilateral/bilateral) of the operation, prosthesis type (for knees, unicondylar/total knee replacement), and Harris Hip Score (HHS) for hip patients and Knee Society Knee and Function Score (KSS) for knee patients.

A modified Charlson’s Comorbidity Index score [28] was calculated for each patient based on the drug reimbursement data. A score of 1 was assigned for heart failure, coronary artery disease, diabetes type I or type II, chronic asthma or other severe obstructive pulmonary disease, dementia, disseminated connective tissue diseases, and rheumatoid arthritis and other comparable conditions. A score of 2 was assigned for uremia requiring dialysis, severe anemia in connection with chronic renal failure, leukemia and other malignant diseases of blood and bone marrow (including malignant diseases of the lymphatic system), and cancer (including breast and prostate cancers, female genital tract cancer, and malignant neoplasms).

Although differences in the trajectories of analgesic consumption among hip and knee replacement patients were reported in a previous analysis [27], in this study, the risk factors for the use of analgesics postoperatively were nearly identical. Therefore, the results for hips and knees are shown together, except for the analyses concerning the effects of laterality, prosthesis type, and hip and knee scores.

The analyses were performed using IBM SPSS Statistics 24. Parametric variables are presented with mean and standard deviation (SD). Student’s *T* test was used to compare parametric variables and chi-squared test was used to compare categorical variables. Multivariable adjusted log-binomial regression was used to calculate adjusted risk ratios (RR_a_) with 95% confidence intervals (CI) for using different types of analgesics. Adjustments in the multivariate model included age, gender, joint, BMI, Charlson Comorbidity Index score, preoperative purchases of antidepressants, and laterality (bilateral/unilateral). *P* values of < 0.05 were considered statistically significant.

### Ethics and registration

The use of drug register data was approved by the Social Insurance Institution of Finland, and permission to use other patient-related data was applied for from the hospital authorities responsible for that data. As this was a retrospective register study, no consent from the Ethical Board or the patients was required.

## Results

The mean age of the study population was 68.7 years (67.6 years for hip patients and 69.7 years for knee patients) and 61.1% were women (53.2% of hip and 67.7% of knee patients). Mean BMI was 29.1 kg/m^2^ (28.2 for hip patients and 29.9 on knee patients). Comorbidities are shown in Table 1.

**Table 1:** Demographic characteristics

	Hip replacement	Knee replacement
Total number, *n*	6238	7501
Age, mean (SD), years	67.59 (10.63)	69.67 (9.52)
Female	53.2%	67.7%
Bilateral operation, *n* (%)	418 (6.7%)	1225 (16.3%)
BMI, mean (SD), kg/m^2^*	28.2 (4.7)	29.9 (4.8)
Charlson Comorbidity Index**		
0	4685 (75.1%)	5239 (69.8%)
1	1128 (18.1%)	1621 (21.6%)
> 2	425 (6.8%)	641 (8.5%)
Diabetes, *n* (%)	473 (7.6%)	744 (9.9%)
Cardiac disease***, *n* (%)	699 (11.2)	904 (12.1%)
Psychotic disorder, *n* (%)	92 (1.5%)	137 (1.8%)
Neurodegenerative disease (Alzheimer or Parkinson), *n* (%)	86 (1.4%)	111 (1.5%)
Pulmonary disease, *n* (%)	373 (6.0%)	619 (8.3%)
Hypertension, *n* (%)	1656 (26.5%)	2491 (33.2%)
History of malignancy, *n* (%)	182 (2.9%)	273 (3.6%)
Epilepsy, *n* (%)	67 (1.1%)	74 (1.0%)
Antidepressant****, *n* (%)	621 (10.0%)	840 (11.2%)
Preoperative analgesic use, *n* (%)		
Any analgesic	2959 (47.4%)	2960 (39.5%)
Acetaminophen	870 (13.9%)	894 (11.9%)
NSAID	2095 (33.6%)	1983 (26.4%)
Opioid	870 (13.9%)	685 (9.1%)
KSS Knee Score, *n* (%)		
Poor (< 60)		3965 (52.9%)
Fair (60–70)		642 (8.6%)
Good or excellent (> 70)		277 (3.7%)
Missing		2617 (34.9%)
KSS Function Score, *n* (%)		
Poor (< 60)		2760 (36.8%)
Fair (60–70)		623 (8.3%)
Good or Excellent (> 70)		1473 (19.6%)
Missing		2645 (35.3%)
Harris Hip Score, *n* (%)		
Poor (< 70)	3801 (60.9%)	
Fair (70–80)	240 (3.8%)	
Good or excellent (> 80)	59 (0.9%)	
Missing	2138 (34.3%)	

*SD* standard deviation, *BMI* body mass index

*Missing on 825 (13.2%) of hip and 997 (13.3%) of knee patients

** Modified Charlson Comorbidity Index

***Coronary artery disease, heart failure, chronic arrhythmia

****Redeemed antidepressant 1 year before surgery

Three months preoperatively, 43.1% of patients redeemed at least one type of analgesic drug, most commonly NSAIDs (29.7%), followed by acetaminophen (12.8%), and opioids (11.3%). One year after surgery, the proportion of patients who redeemed at least one type of analgesic drug decreased to 26.1%, and NSAIDs were still the most common (15.5%), followed by acetaminophen (10.1%), and opioids (6.7%).

### Patient characteristics

A higher proportion of older patients redeemed any analgesic drug 1 year postoperatively (29.0% of patients aged > 75 years) than younger patients (26.2% of patients aged 65–75 years and 23.7% of patients aged < 65 years; *p* < 0.001) (Additional file 1). In the adjusted model, patients older than 75 years (reference < 65 years) had higher RR_a_ for the use of any analgesic drug (RR_a_ 1.2 [95% CI 1.1–1.3]) and acetaminophen (2.2 [1.9–2.5]) and lower RR_a_ for the use of NSAIDs (RR_a_ 0.77 [0.68–0.86]) (Table 2). A higher proportion of women (28.8%) than men (22.0%) redeemed any analgesic drug postoperatively (*p* < 0.001). In the adjusted model, women had higher RR_a_ for the use of any analgesics, acetaminophen, and NSAIDs, but not opioids (Table 2).

**Table 2:** Multivariable adjusted risk ratios (RR_a_) for analgesic consumption

	Preoperative (0–3 months)	Postoperative (9–12 months)			
	Any analgesic**	Any analgesic**	Acetaminophen	NSAID	Opioid
Age, years					
< 65	1	1	1	1	1
65–75	1.02 (0.97–1.1)	**1.1 (1.03–1.2)**	**1.4 (1.2–1.7)**	0.94 (0.85–1.03)	1.03 (0.87–1.2)
> 75	1.01 (0.95–1.1)	**1.2 (1.1–1.3)**	**2.2 (1.9–2.5)**	**0.77 (0.68–0.86)**	1.06 (0.89–1.3)
Female gender	**1.1 (1.1–1.2)**	**1.2 (1.1–1.3)**	**1.4 (1.2–1.6)**	**1.2 (1.1–1.3)**	1.1 (0.98–1.3)
BMI, kg/m^2^					
< 25	1	1	1	1	1
25 < 30	1.04 (0.98–1.1)	1.1 (0.97–1.2)	1.01 (0.87–1.2)	1.1 (0.96–1.2)	1.01 (0.83–1.2)
30–35	**1.1 (1.05–1.2)**	**1.1 (1.04–1.2)**	**1.2 (1.03–1.4)**	1.1 (0.99–1.3)	1.1 (0.91–1.4)
> 35	**1.3 (1.2–1.4)**	**1.4 (1.3–1.6)**	**1.6 (1.4–2.0)**	**1.5 (1.3–1.7)**	**1.4 (1.1–1.8)**
BMI, constant, per increase of 1 kg/m^2^	**1.02 (1.01–1.02)**	**1.02 (1.02–1.02)**	**1.03 (1.02–1.04)**	**1.02 (1.01–1.03)**	**1.02 (1.01–1.03)**
Charlson Comorbidity Index***					
0	1	1	1	1	1
1	**1.1 (1.00–1.1)**	**1.2 (1.1–1.3)**	**1.3 (1.2–1.5)**	1.04 (0.94–1.2)	**1.3 (1.1–1.5)**
2 or more	**1.1 (1.01–1.2)**	**1.2 (1.1–1.4)**	**1.4 (1.2–1.6)**	1.02 (0.87–1.2)	**1.8 (1.5–2.2)**
Diabetes					
No	1	1	1	1	1
Yes, but without insulin medication	0.99 (0.94–1.04)	1.1 (0.99–1.3)	1.01 (0.90–1.1)	1.02 (0.93–1.1)	1.1 (0.95–1.3)
Yes, with insulin medication	1.02 (0.93–1.1)	0.98 (0.88–1.1)	0.95 (0.79–1.2)	0.89 (0.74–1.1)	1.1 (0.86–1.4)
Cardiac disease****	**0.86 (0.80–0.93)**	0.94 (0.85–1.04)	0.96 (0.81–1.1)	**0.77 (0.65–0.91)**	1.2 (0.94–1.4)
Psychotic disorder	1.1 (0.92–1.2)	1.1 (0.94–1.3)	1.2 (0.93–1.7)	0.77 (0.55–1.1)	1.4 (0.96–1.9)
Neurodegenerative disease*****	1.1 (0.96–1.3)	1.03 (0.84–1.3)	**1.4 (1.1–1.9)**	0.88 (0.59–1.3)	0.62 (0.35–1.1)
Chronic lung disease	1.1 (0.98–1.2)	1.1 (0.97–1.2)	1.1 (0.89–1.3)	**1.2 (1.03–1.4)**	1.1 (0.86–1.4)
Hypertension	1.01 (0.97–1.1)	1.1 (0.997–1.1)	1.04 (0.93–1.2)	1.03 (0.93–1.1)	1.1 (0.98–1.3)
History of malignancy	1.1 (0.96–1.3)	1.1 (0.89–1.3)	1.00 (0.74–1.4)	1.2 (0.86–1.6)	0.97 (0.68–1.4)
Epilepsy	1.1 (0.91–1.3)	1.2 (0.99–1.6)	1.3 (0.86–2.0)	0.93 (0.61–1.4)	**1.9 (1.3–3.0)**
Knee replacement vs hip replacement	**0.77 (0.74–0.81)**	**1.2 (1.1–1.3)**	**1.1 (1.01–1.3)**	**1.2 (1.1–1.3)**	**1.2 (1.1–1.4)**
Preoperative analgesic use******	–	**2.6 (2.5–2.8)**	**2.7 (2.4–3.0)**	**2.7 (2.4–2.9)**	**3.9 (3.3–4.6)**

RR (95% CI) adjusted with age (continuous), gender, joint, Charlson Comorbidity Index, BMI (continuous), laterality (unilateral vs bilateral), and use of antidepressant 1 year before surgery

**Acetaminophen, NSAID, opioid

***Modified Charlson Comorbidity Index

****Coronary artery disease, heart failure, chronic arrhythmia

*****Alzheimer’s or Parkinson’s disease

******Use of any analgesic drug (acetaminophen, NSAID, and/or opioid) preoperatively

### Comorbidities

**Fig. 2:**
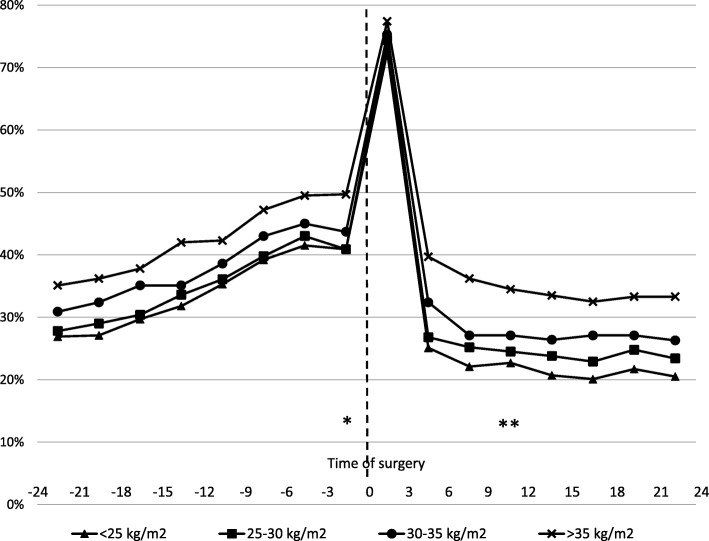
The proportions of patients with any analgesic drugs in 3 months intervals according to BMI. Legend: Time periods used in the adjusted analyses are marked with * (preoperative) and ** (postoperative)

High body mass index (BMI) preoperatively was associated with the use of any analgesic drugs (Fig. 2). One year postoperatively, 22.7% of patients with BMI < 25, 24.5% of patients with BMI 25–30, 27.7% of patients with BMI 30–35, and 29.0% of patients with BMI > 35 redeemed analgesics (*p* < 0.001). In the adjusted model, patients with BMI > 35 kg/m^2^ (reference < 25 kg/m^2^) had higher RR_a_ for the use of any analgesic drug, acetaminophen, NSAIDs, and opioids (Table 2). BMI was also associated with the higher RR_a_ of drug use as a continuous variable (Table 2).

A high Charlson Comorbidity Index (CCI) score was associated with any analgesic drug use both pre- and postoperatively. Postoperatively, 24.1% of patients with a score of 0, 30.7% of patients with a score of 1, and 33.1% of patients with a score of 2 or more redeemed any analgesic drug (Additional file 1). In the adjusted model, a CCI score of 2 or more was associated with higher RR_a_ for the use of any analgesic drugs (RR_a_ 1.2 [1.1–1.4]), acetaminophen (RR_a_ 1.4 [1.2–1.6]), and opioids (RR_a_ 1.8 [1.5–2.2]). The results were similar when patients with a history of malignancy were excluded (data not shown).

Although many of the individual comorbidities were associated with a greater proportions of users of any analgesic drug postoperatively (Additional file 1), after adjustments, only chronic lung disease was associated with the use of NSAIDs (RR_a_ 1.2 [1.03–1.4]), and epilepsy was associated with the use of opioids (RR_a_ 1.9 [1.3–3.0]). Cardiac disease, however, was associated with lower RR_a_ for using NSAIDs (RR_a_ 0.77 [0.65–0.91]) (Table 2). Neurodegenerative disease was associated with higher RR_a_ for using acetaminophen (RR_a_ 1.4 [1.1–1.9]).

### Other clinical factors

A higher proportion of knee patients than hip replacement patients used any analgesic drug 9–12 months postoperatively (28.8% vs 23.0%, *p* < 0.001) and differences in the use of any analgesic drugs, acetaminophen, NSAIDs, and opioids remained the same after adjustments (Table 2, Additional file 1). Patients who had redeemed any analgesic drugs preoperatively also redeemed them more often postoperatively (40.6% vs 15.2%, *p* < 0.001) and similar results were seen in the adjusted model (Table 2, Additional file 1).

#### Joint-specific issues

Patients receiving bilateral hip or knee replacements tended to use fewer analgesics postoperatively (Additional file 1). However, in the adjusted analyses, statistically significant differences were only observed for acetaminophen both after hip and knee replacement and opioids after knee replacement (Table 3). A higher proportion of total knee replacement patients than unicompartmental knee replacement patients used any analgesic drugs (29.1% vs 23.5%, *p* = 0.007) (Additional file 1). In the adjusted model, patients with unicompartmental knee replacement had lower RR_a_ for the use of acetaminophen (RR_a_ 0.45 [0.28–0.72]) and opioids (0.52 [0.32–0.85]) after surgery (Table 3). Both before hip and knee replacement, a better preoperative situation, as measured with the Harris Hip Score and Knee Society Score, respectively, was associated with lower preoperative user rate of any analgesics (Table 3). However, preoperative clinical state, based on HHS/KSS, had no statistically significant association with postoperative overall analgesic use (Table 3), although “fair” KSS Function score was associated with lower RR_a_ for the use of acetaminophen and “good or excellent” score with lower RR_a_ for use of opioids.

**Table 3:** Multivariable adjusted risk ratios (RR_a_) for analgesic consumption according to clinical factors

	Preoperative (0–3 months)	Postoperative (9–12 months)			
	Any analgesic**	Any analgesic**	Acetaminophen	NSAID	Opioid
**Hip replacement**					
Laterality:bilateral	1.1 (0.999–1.2)	0.82 (0.64–1.04)	**0.34 (0.17–0.68)**	1.02 (0.77–1.3)	0.67 (0.38–1.2)
Harris Hip Score***					
Poor (< 70)	1	1	1	1	1
Fair (70–80)	**0.77 (0.63–0.94)**	0.78 (0.54–1.1)	0.65 (0.29–1.4)	0.91 (0.61–1.3)	0.58 (0.22–1.6)
Good or excellent (> 80)	**0.49 (0.30–0.82)**	0.60 (0.28–1.3)	1.36 (0.05–2.5)	0.72 (0.31–1.7)	–
**Knee replacement**					
Laterality:bilateral	**1.1 (1.02–1.2)**	**0.86 (0.77–0.96)**	**0.77 (0.62–0.96)**	0.91 (0.78–1.1)	**0.56 (0.42–0.76)**
UKA (vs TKA)	**0.72 (0.61–0.84)**	0.83 (0.69–1.002)	**0.45 (0.28–0.72)**	0.99 (0.79–1.2)	**0.52 (0.32–0.85)**
KSS Knee Score***					
Poor (< 60)	1	1	1	1	1
Fair (60–70)	**0.81 (0.71–0.92)**	0.94 (0.82–1.1)	0.83 (0.62–1.1)	1.03 (0.86–1.2)	0.73 (0.50–1.05)
Good or excellent (> 70)	**0.78 (0.64–0.95)**	0.88 (0.70–1.1)	0.81 (0.52–1.3)	0.94 (0.70–1.3)	0.82 (0.49–1.4)
KSS Function Score***					
Poor (< 60)	1	1	1	1	1
Fair (60–70)	0.94 (0.84–1.1)	1.03 (0.90–1.2)	**1.3 (1.02–1.7)**	0.90 (0.73–1.1)	0.91 (0.65–1.3)
Good or excellent (> 70)	**0.90 (0.82–0.99)**	0.89 (0.80–1.00)	0.95 (0.75–1.2)	0.97 (0.83–1.1)	**0.73 (0.55–0.97)**

RR (95% CI) adjusted with age (continuous), gender, Charlson Comorbidity Index, BMI (continuous), laterality (unilateral vs bilateral), and use of antidepressant 1 year before surgery

**Acetaminophen, NSAID, opioid

***Preoperative HHS missing on 2138 (34.3%) patients and KSS Knee Score on 2617 (34.9%) patients and KSS Function Score on 2645 (35.3%) patients

## Discussion

This large study of an unselected osteoarthritis population undergoing hip or knee replacement found that higher age, female gender, obesity, number of comorbidities (according to the modified Charlson Comorbidity Index score), preoperative use of analgesics, and unilateral knee replacement (compared to simultaneous bilateral procedure) were associated with a higher probability of using analgesic drugs 1 year after surgery, whereas individual comorbid conditions had little or no effect. The associations related to obesity, gender, operated joint, and preoperative use of analgesics were similar for different types of analgesic agents whereas the associations with different comorbid conditions were more mixed. Moreover, the results suggest that NSAIDs are avoided in patients with multimorbidity, especially cardiac disease, and in older patients, which is supported by current guidelines [29]. The present study expands on the earlier literature by analyzing the impact of several comorbidities and clinical factors on the postoperative consumption of all analgesic drugs and by including not only NSAIDs and opioids, but also acetaminophen.

Our main finding is that obesity was associated with a higher risk ratio for the consumption of all the studied analgesic drugs both pre- and postoperatively. Previously, a higher BMI has been shown to predict NSAID use after THA and TKA [18–20] and opioid use after TKA [14, 19]. Our study shows that obesity predicts the use of all analgesic drugs (acetaminophen, NSAIDs, and opioids) after hip or knee replacement. Similar to the findings of earlier studies [18–20], drug use was more frequent especially when patient BMI exceeded 35 kg/m^2^. Obese patients report more pain after joint replacement [7, 18], which is one possible explanation for this finding. Obesity has also been associated with analgesic use in the general population [30, 31].

The number of comorbidities measured with CCI score predicted the use of acetaminophen and opioids, but not NSAIDs. Earlier, Hansen et al. [13] reported an association between higher CCI score and chronic opioid use after TKA. We were, however, unable to provide a good explanation for this finding. A history of malignancy, a component of the CCI with a score of 2, could be one possible explanation, but a CCI score of 1 also predicted analgesics use, and the results considering CCI in the logistic regression were identical, even when patients with a history of malignancy were excluded. Additionally, a history of malignancy was not independently associated with analgesic use.

Out of the separate comorbidities, patients with neurodegenerative disorders used acetaminophen (but not NSAIDs or opioids) more often. This may be related to the adverse events of opioids and NSAIDs in long-term use in the elderly [22–24, 32, 33]. As expected, patients with cardiac disease used fewer NSAIDs than other patients [23, 24]. Patients with epilepsy use more opioids in the general population [34], possibly due to a higher prevalence of painful conditions, and we found a similar finding in joint replacement recipients. Interestingly, although diabetes is associated with persistent postoperative pain [7], the disease was not associated with analgesic consumption, especially when their higher BMIs were taken into account [7]. Earlier, controversial results have been reported. Namba et al. [14] reported an association with diabetes and increased use of opioids after knee replacement (OR 1.03 [95% CI 1.01–1.05]), whereas in two other studies [9, 15], no differences were found after knee or hip replacement regarding diabetes. These differences may be explained by over twofold higher prevalence of diabetes in the study by Namba et al. than in the other two and the present study.

Patients who underwent knee surgery used acetaminophen, NSAIDs, and opioids more often than patients who underwent hip surgery [12, 15, 20]. Patients with simultaneous bilateral knee surgery used more analgesics preoperatively (compared to unilateral operation) but used fewer opioids and acetaminophen postoperatively. These findings may reflect patient selection bias as those patients with previous opioid use or signs of a history of pain sensitization are not candidates for simultaneous bilateral knee arthroplasty. It is not known whether a simultaneous or staged bilateral operation should be preferred in bilateral osteoarthritis [35], and a comparison of these procedures was not possible in the present study due to the chosen exclusion criteria (another joint replacement 2 years before or after index surgery; therefore, patients with staged bilateral operations were excluded). Similar to the findings of earlier studies [9, 21], UKA (compared to TKA) was associated with lower RR_a_ for the use of opioids and acetaminophen. Interestingly, preoperative clinical state, based on HHS/KSS, was not associated with postoperative overall analgesic use although better KSS Function score was associated with lower RR_a_ for the use of acetaminophen and opioids.

Preoperative pain has been associated with postoperative analgesic use [9], and although it is a component of KSS Knee Score and HHS, these were not associated with postoperative analgesic use. Preoperative pain was not possible to analyze separately in this study.

In line with earlier studies [12, 14, 15, 21, 26], preoperative analgesic use was associated with greater postoperative opioid use. In this study, we also found a similar association with the use of acetaminophen and NSAIDs. Furthermore, opioid consumption was greater in patients with higher CCI score, epilepsy, obesity (BMI > 35 kg/m^2^), knee vs hip surgery, unilateral knee replacement vs bilateral operation, TKA vs UKA, and those who used analgesics preoperatively. Previously, increased opioid consumption after hip and knee replacement has been associated with TKA vs THA, preoperative opioid or other analgesic use, psychiatric disorders (especially depression/anxiety), tobacco use, cardiac disease, younger age, greater affected joint pain, other pain sites, TKA vs UKA, use of walking aids, and female gender [9, 12–15, 17–19, 21, 26]. In addition, obesity and the number of comorbidities (based on CCI score) have been associated with increased opioid use after knee replacement [14, 19]. The new findings in this study were that obesity, epilepsy, and overall comorbidity (based on modified CCI score) were associated with greater opioid use after both hip and knee replacement.

To the best of our knowledge, factors associated with postoperative consumption of acetaminophen have not been analyzed in previous literature. In this study, its use was in general mostly associated with the same factors as the overall analgesic use. This is expected as acetaminophen has been considered the basis of pain management [36] and it was the most common analgesic also in this study. Increased use in patients with higher age, number of comorbidities, and those with neurodegenerative disease is likely explained by avoidance of NSAID and opioid use in these patient groups.

The main strength of our study is the inclusion of all analgesic drug groups (acetaminophen, NSAID, opioid) in a large sample of unselected joint replacement patients. We included only primary joint replacements and only osteoarthritis as an indication for surgery. Other indications were excluded because, for example, patients with rheumatoid arthritis tend to have polyarticular involvement more often, which serves to hamper the analysis of the use of analgesic drugs. Patients with revision or other joint replacement during the follow-up period were excluded because the perioperative peak in the consumption of analgesic drugs related to the latter operation would have been a potentially confounding factor considering the analysis of postoperative analgesic use after index surgery. According to our previous analysis [27], 9–12 months after surgery appears to be a suitable time point for analyzing the level of postoperative analgesic use. Because the use patterns are similar after hip and knee replacement and also among different comorbid conditions, the materials could be analyzed as a whole to increase statistical power. All operations were performed in a single orthopedic hospital with standardized perioperative care, anesthesia, and analgesia. Medication data include all the prescribed medications that were dispensed in Finland. The presence of comorbidities was based on a nationwide Reimbursement Register that covers the most important comorbidities, although a clear limitation is that the register does not cover mental health problems other than psychotic disorders. However, we included the use of antidepressants in the adjusted model, along with several other possible confounders.

The limitations of the study include the lack of information on OTC drugs and the fact that in a register study based on redeemed prescriptions, it is not possible to find out the indication for the analgesic drug (index joint or other pain sites) nor whether the patient has taken the drug or not. For these reasons, the actual use of acetaminophen and NSAIDs (that are available OTC) might be higher than reported here, but it is unlikely that this would have affected the use patterns or associated factors. In the general population, higher analgesic use has been found on women, elderly, smokers, obese, and patients with chronic diseases or with several medications [30, 31, 37], which should be noted in the interpretation of the results since indication for the analgesic is not known. Although patients with major complications leading to revision were excluded, we were not able to analyze all postoperative complications. Moreover, preoperative pain, the intensity of pain, and the prevalence of persistent pain were not examined in this study.

## Conclusions

In conclusion, this study adds to the earlier literature that female gender, obesity, number of comorbidities (according to the modified Charlson Comorbidity Index score), preoperative use of analgesics, and unilateral knee replacement (compared to simultaneous bilateral procedure) were associated with the probability of using analgesic drugs 1 year after joint replacement. On the other hand, single comorbidities, such as diabetes, and preoperative clinical state were not associated with overall analgesic use. The strongest predictors of increased postoperative use of analgesics were obesity (especially BMI > 35 kg/m^2^) and the preoperative use of analgesics. These results were essentially similar for all types of analgesics including acetaminophen which seemed to replace NSAIDs in older patients and in patients with multimorbidity. It is remarkable that older age and higher number of comorbidities predicted analgesic use despite these patients also being the most vulnerable to adverse drug events. Clinicians should therefore inform patients with obesity (especially BMI > 35 kg/m^2^) about the elevated risk for prolonged use of analgesics (including opioids) after surgery.

## Supplementary information

**Additional file 1.** Proportions of patients with any analgesic drug*.

## Data Availability

National legislation and data protection regulations do not allow the sharing of the patient-level materials of this study. Summarized data (such as patient numbers) can be provided by the corresponding author upon request.

## Abbreviations

ATC code: Anatomical Therapeutic Chemical code
BMI: Body mass index
CCI score: Charlson’s Comorbidity Index score
CI: Confidence interval
NSAID: Non-steroidal anti-inflammatory drug
RR: Risk ratio
OTC drug: Over-the-counter drug
SD: Standard deviation
SII: Social Insurance Institution
THA: Total hip arthroplasty
TKA: Total knee arthroplasty
UKA: Unicondylar knee arthroplasty
