# Supplementary material for: Predictors of the use of analgesic drugs 1 year after joint replacement: a single-center analysis of 13,000 hip and knee replacements
Source: Arthritis Res Ther. 2020 Apr 21;22:89. doi: 10.1186/s13075-020-02184-1 (PMC7175525; doi:10.1186/s13075-020-02184-1)
Supplement: Supplementary file 1 — Additional file 1. Proportions of patients with any analgesic drug*. [file 13075_2020_2184_MOESM1_ESM.docx]

| **Additional file 1.** Proportions of patients with any analgesic drug* | | | |
| --- | --- | --- | --- |
|  | | Preoperative (0-3 months)  N (%) | Postoperative (9-12 months)  N (%) |
| All patients | | 5919 (43,1%) | 3591 (26,1%) |
| Age, y | |  |  |
|  | <65 | 2108 (43,8%) | 1141 (23,7%) |
|  | 65-75 | 2092 (43,5%) | 1258 (26,2%) |
|  | >75 | 1719 (41,8%) | 1192 (29,0%) |
|  | p-value | 0.125 | <0.001 |
| Gender | |  |  |
|  | Female | 3766 (44,9%) | 2417 (28,8%) |
|  | Male | 2153 (40,3%) | 1174 (22,0%) |
|  | p-value | <0.001 | <0.001 |
| BMI, kg/m^2^ | |  |  |
|  | <25 | 954 (41,0%) | 529 (22,7%) |
|  | 25-29.9 | 2009 (40,8%) | 1207 (24,5%) |
|  | 30-35 | 1401 (43,7%) | 871 (27,2%) |
|  | >35 | 683 (49,8%) | 473 (34,5%) |
|  | p-value | <0.001 | <0.001 |
| Charlson comorbidity index | |  |  |
|  | 0 | 4204 (42,4%) | 2394 (24,1%) |
|  | 1 | 1231 (44,8%) | 844 (30,7%) |
|  | 2 or more | 484 (45,4%) | 353 (33,1%) |
|  | p-value | 0.022 | <0.001 |
| Diabetes | |  |  |
|  | No | 3889 (42,8%) | 2230 (24,5%) |
|  | Yes, but without insulin medication | 1470 (42,8%) | 990 (28,8%) |
|  | Yes, with insulin medication | 560 (46,0%) | 371 (30,5%) |
|  | p-value | 0.096 | <0.001 |
| Cardiac disease** | |  |  |
|  | No | 5280 (43,5%) | 3116 (25,7%) |
|  | Yes | 639 (39,9%) | 475 (29,6%) |
|  | p-value | 0.006 | 0.001 |
| Psychotic disorder | |  |  |
|  | No | 5799 (42,9%) | 3505 (25,9%) |
|  | Yes | 120 (52,4%) | 86 (37,6%) |
|  | p-value | 0.004 | <0.001 |
| Chronic lung disease | |  |  |
|  | No | 5438 (42,7%) | 3254 (25,5%) |
|  | Yes | 481 (48,5%) | 337 (34,0%) |
|  | p-value | <0.001 | <0.001 |
| Neurodegenerative disease | |  |  |
|  | No | 5820 (43,0%) | 3517 (26,0%) |
|  | Yes | 99 (50,3%) | 74 (37,6%) |
|  | p-value | 0.041 | <0.001 |
| Hypertension | |  |  |
|  | No | 4096 (42,7%) | 2365 (24,7%) |
|  | Yes | 1823 (44,0%) | 1226 (29,6%) |
|  | p-value | 0.172 | <0.001 |
| History of malignancy | |  |  |
|  | No | 5706 (43,0%) | 3445 (25,9%) |
|  | Yes | 213 (46,8%) | 146 (32,1%) |
|  | p-value | 0.102 | 0.003 |
| Epilepsy | |  |  |
|  | No | 5854 (43,1%) | 3545 (26,1%) |
|  | Yes | 65 (46,1%) | 46 (32,6%) |
|  | p-value | 0.467 | 0.078 |
|  |  |  |  |
| Knee vs hip surgery | |  |  |
|  | Knee replacement | 2960 (39.5%) | 2158 (28.8%) |
|  | Hip replacement | 2959 (47.4%) | 1433 (23.0%) |
|  | p-value | p<0.001 | p<0.001 |
|  |  |  |  |
| Preoperative analgesic use*** | |  |  |
|  | No | - | 1185 (15.2%) |
|  | Yes | - | 2406 (40.6%) |
|  | p-value |  | p<0.001 |
|  |  |  |  |
| **Hip replacement** | |  |  |
| Laterality | |  |  |
|  | Unilateral | 2754 (47.3%) | 1364 (23.4%) |
|  | Bilateral | 205 (49.0%) | 69 (16.5%) |
|  | p-value | p=0.495 | p=0.001 |
| Harris hip score (miss 2138) | |  |  |
|  | Poor (<70) | 1735 (45,6%) | 800 (21,0%) |
|  | Fair (70-80) | 80 (33,3%) | 32 (13,3%) |
|  | Good or excellent (>80) | 12 (20,3%) | 6 (10,2%) |
|  | p-value | <0.001 | 0.002 |
|  | |  |  |
| **Knee replacement** | |  |  |
| Laterality | |  |  |
|  | Unilateral | 2437 (38.8%) | 1865 (29.7%) |
|  | Bilateral | 523 (42.7%) | 293 (23.9%) |
|  | p-value | p=0.011 | p<0.001 |
| UKA vs TKA | |  |  |
|  | UKA | 148 (29.7%) | 117 (23.5%) |
|  | TKA | 2812 (40.2%) | 2041 (29.1%) |
|  | p-value | <0.001 | 0.007 |
| KSS Knee Score (miss 2617) | |  |  |
|  | Poor (<60) (n=3695) | 1562 (39,4%) | 1081 (27,3%) |
|  | Fair (>=60, <70) (n=642) | 203 (31,6%) | 168 (26,2%) |
|  | Good (70-80) (n=221) | 68 (30,8%) | 54 (24,4%) |
|  | Excellent (>80) (n=56) | 17 (30,4%) | 13 (23,2%) |
|  | p-value | <0.001 | 0.681 |
| KSS Function Score (miss 2645) | |  |  |
|  | Poor (<60) (n=2760) | 1095 (39,7%) | 785 (28,4%) |
|  | Fair (>=60, <70) (n=623) | 229 (36,8%) | 176 (28,3%) |
|  | Good (70-80) (n=1151) | 406 (35,3%) | 274 (23,8%) |
|  | Excellent (>80) (n=322) | 95 (29,5%) | 66 (20,5%) |
|  | p-value | 0.001 | 0.001 |

UKA, unicompartmental knee arthroplasty; TKA, total knee arthroplasty

*acetaminophen, NSAID, or opioid

**coronary artery disease, hearth failure, chronic arrhythmia

*** Use of any analgesic drug (acetaminophen, NSAID, or opioid) preoperatively
